# Transcutaneous auricular vagus nerve stimulation ameliorates adolescent depressive‐ and anxiety‐like behaviors via hippocampus glycolysis and inflammation response

**DOI:** 10.1111/cns.14614

**Published:** 2024-02-15

**Authors:** Lan Sun, Shixiang Ma, Yun Yu, Xiangji Li, Qianwen Wei, Li Min, Peijing Rong

**Affiliations:** ^1^ Institute of Acupuncture and Moxibustion, China Academy of Chinese Medical Sciences Beijing China; ^2^ Department of Retroperitoneal Tumor Surgery Peking University International Hospital Beijing China; ^3^ School of Life Science and Technology Xi'an Jiaotong University Xi'an China; ^4^ State Key Laboratory for Digestive Health, National Clinical Research Center for Digestive Diseases, Department of Gastroenterology Beijing Friendship Hospital, Capital Medical University Beijing China; ^5^ School of Acupuncture‐Moxibustion and Tuina Beijing University of Chinese Medicine Beijing China; ^6^ Institute of Basic Research in Clinical Medicine China Academy of Chinese Medical Sciences Beijing China

**Keywords:** adolescent depression, different brain regions, transcriptome, transcutaneous auricular vagus nerve stimulation

## Abstract

**Background:**

Transcutaneous auricular vagus nerve stimulation (taVNS) is a crucial neuromodulation therapy for depression, yet its molecular mechanism remains unclear. Here, we aim to unveil the underlying mechanisms of antidepression by systematically evaluating the change of gene expression in different brain regions (i.e., hippocampus, anterior cingulate cortex, and medial prefrontal cortex).

**Methods:**

The adolescent depression rat model was established by chronic unpredictable mild stress (CUMS), followed by the taVNS treatment for 3 weeks. The open field test (OFT), forced swimming test (FST), elevated plus maze test (EPM), and new object recognition (NOR) test were used to evaluate depressive‐ and anxiety‐like behaviors. Gene expression analysis of three brain regions was conducted by RNA sequencing (RNA‐seq) and further bioinformatics methods.

**Results:**

The depressive‐ and anxiety‐like behaviors in CUMS‐exposed rats were manifested by decreased spontaneous locomotor activity of OFT, increased immobility time of FST, increased entries and time in the closed arms of EPM, and decreased new object index of NOR. Furthermore, CUMS exposure also led to alterations in gene expression within the hippocampus (HIP), anterior cingulate cortex (ACC), and medial prefrontal cortex (mPFC), suggesting a potential link between adolescent stress and pathological changes within these brain regions. TaVNS could significantly ameliorate depressive‐ and anxiety‐like behaviors. Its effects on these three brain regions were found related to regulation of the metabolism, and there were some brain region‐specific findings. Compared with ACC and mPFC, taVNS has a more concrete effect on HIP by regulating the inflammation response and glycolysis.

**Conclusion:**

taVNS is capable of ameliorating adolescent depressive‐ and anxiety‐like behaviors by regulating plenty of genes in the three brain regions. Suppressed level of inflammatory response and enhanced glycolysis manifests the dominant role of taVNS in HIP, which provides a theoretical foundation and data support for the molecular mechanism of antidepression by taVNS.

## Graphical abstract

Transcutaneous auricular vagus nerve stimulation has the capability to improve depressive‐ and anxiety‐like behaviors in adolescent rats by regulating numerous genes in three key brain regions associated with emotions.

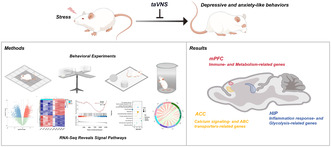

## 1 INTRODUCTION

Adolescence constitutes a pivotal period for the maturation of neurocognitive processes, during which stress impacts brain development and results in an augmented vulnerability to mental disorders in later life.^1^, ^2^ Exposure to early life adversity (ELA) is a well‐established predictor of stress‐related psychopathologies such as depression.^3^, ^4^, ^5^, ^6^, ^7^ The link between ELA and aberrant cognitive behaviors in maturity is well supported by preclinical models of peri‐adolescent (early‐to‐late adolescence) populations.^8^, ^9^ Various forms of social adversity during adolescence, including bullying, sexual and emotional abuse, household dysfunction, assault, social isolation, and other unpredictable chronic traumatic events, significantly increase neuropsychiatric outcomes in adolescents as well as in adults.^8^, ^10^

Depression is the most common mental health disorder among youth,^11^ with an estimated incidence of 4%–5% in the teenage population worldwide.^12^ Symptoms of depression in adolescence are likely to recur throughout life without proper intervention and may lead to serious interpersonal concerns and self‐injurious behavior. Furthermore, depression ranks as the second most prevalent cause of mortality worldwide among individuals aged 15–29.^13^ Adolescents with depression were often treated with dopamine–serotonin receptor antagonists and other psychotropic medications yet more or less with adverse effects.^14^, ^15^ In addition, about 40% of them fail the treatment either with antidepressant medication or evidence‐based psychotherapy.^16^, ^17^, ^18^ Therefore, it is imperative to urgently develop non‐pharmacological intervention strategies for youth depression.

The vagus nerve, which serves as the neural trunk of the parasympathetic division of the autonomic nervous system, establishes connections between the central nervous system (CNS) and peripheral organs, including but not limited to the spleen, heart, liver, and gastrointestinal tract.^19^, ^20^ With the advancement of electromagnetic medicine, vagus nerve stimulation (VNS) emerges as an adjunctive therapy for treatment‐resistant depression and epilepsy. Although VNS has been approved for neuromodulation therapy, potential side effects, such as surgical complications, dyspnea, and pain, largely restrict its clinical application.^21^

The sole afferent branch of the vagus nerve that can be observed on the body surface is the auricular branch of vagus nerve (ABVN),^22^, ^23^ providing a non‐invasive pathway for VNS‐based treatments. Following extensive peripheral projection, the afferent fibers of the ABVN transmit information to the nucleus tractus solitarius (NTS), which subsequently projects directly or indirectly to various brain regions, including the dorsal raphe nucleus (DRN), locus coeruleus (LC), hippocampus (HIP), hypothalamus, prefrontal cortex (PFC), etc.^24^ Accordingly, non‐invasive transcutaneous auricular VNS (taVNS) has been proposed to treat mental illness and neurodevelopmental disorders.^25^, ^26^, ^27^

Previously, we found that taVNS greatly altered Hamilton Depression Rating Scale (HAMD) scores and default‐mode brain network connectivity in depressed patients.^28^, ^29^ Also, taVNS produced brain activity changes through the ABVN pathway in those patients, showing great potential as an effective and safe neuromodulation alternative treatment for mild and moderate depressive disorder. However, the underlying mechanism remains unrevealed.

Depression is now regarded as being initiated by multiple brain areas, including the hippocampus (HIP), anterior cingulate cortex (ACC), and medial prefrontal cortex (mPFC) associated with the regulation of emotion and cognition.^30^, ^31^ Therefore, it is crucial to depict brain region‐specific gene expression profiles and identify key regulatory genes to elaborate the molecular mechanisms of how taVNS treats depression. Accordingly, we conducted RNA‐sequencing (RNA‐seq) analyses on a diverse range of mood‐related brain regions, including the hippocampus (HIP), anterior cingulate cortex (ACC), and medial prefrontal cortex (mPFC). We identified corresponding alterations in gene expression to investigate the antidepressant effects of taVNS using a chronic unpredictable mild stress (CUMS) model of adolescent depression in rats.

## 2 MATERIALS AND METHODS

### 2.1 Animals and experimental procedure

A total of 30 specific pathogen‐free (SPF) male postnatal day (PND) 21 Sprague–Dawley rats (50 ± 10 g) were supplied by the Laboratory Animal Center of Academy of Military Medical Sciences (Beijing, P. R. China). The animals were housed in standardized laboratory conditions, with four to five animals per cage. These conditions included controlled temperature (20–25°C), a 12‐h light–dark cycle starting at 7:00 a.m., and constant humidity (55 ± 2%). During the acclimatization period of 7 days before the follow‐up experiments, the animals had free access to food and water, except on food restriction days during behavioral tests. The experiment was conducted with minimal noise throughout the entire process to prevent any disruptions. All experimental procedures were in strict accordance with the guidelines of Care and Use of Laboratory Animals of the Ministry of Science and Technology of the People's Republic of China, and were approved by the Ethics Committee of the Institute of Acupuncture and Moxibustion, China Academy of Chinese Medical Sciences (Permit No. Y2023‐03‐10‐03).

**FIGURE 1:**
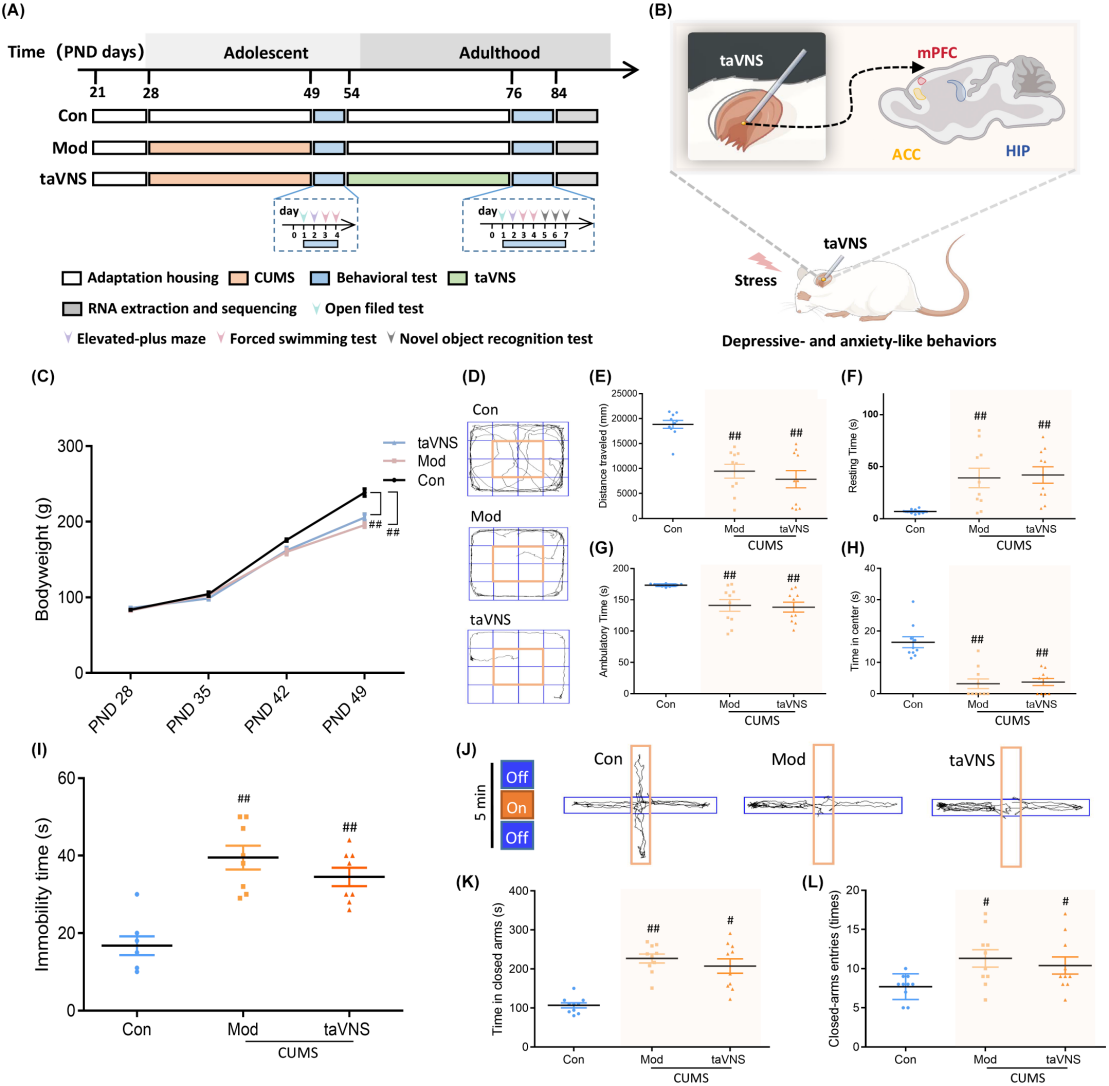
Design of taVNS treatment in adolescent depression rats and their behavioral tests at baseline. (A) The diagram of taVNS treatment procedures. (B) Schematic diagram of the experimental procedure. The receptive area of taVNS is located in the bilateral auricular concha, which corresponds to the distribution area of the auricular branch of the vagus nerve. (C) The effects of CUMS on the body weight at different time points. (D) Movement tracks of rats in different groups in OFT. (E) Comparison of the distance traveled by rats in OFT. (F) Comparison of the resting time in OFT. (G) Comparison of the ambulatory time in OFT. (H) Comparison of the time in center in OFT. (I) The effects of CUMS in FST. (J) Movement tracks of rats in different groups in EPM. (K) Comparison of the time in closed arms in EPM. (L) Comparison of the closed arms entries in EPM. Data were expressed as the mean ± SD (*n* = 10 per group); #*p* < 0.05 versus the control group, ##*p* < 0.01 versus the control group.

Rats were randomly divided into three groups (10 in each group): control group (Con), chronic unpredictable mild stress group (Mod), and taVNS group (taVNS). Except for the Con group, all other two groups underwent the CUMS procedure in a single cage. Following 3 weeks of animal modeling (PND 28–49) and 3 weeks of taVNS intervention (PND 54–75), rats from each group were conducted behavioral tests on PND 50 and PND 76, respectively. As shown in Figure 1A, open‐field test (OFT), elevated plus maze (EPM), force swimming test (FST) and new object recognition (NOR) were carried out on all rats to assess emotion and cognitive function. All the behavioral tests were conducted at 24‐h intervals. In the end, all rats were sacrificed during the adulthood period (PND 84) days. The timeline of procedures is also shown in Figure 1A.

### 2.2 Chronic unpredictable mild stress paradigm

The CUMS procedure was conducted according to the previously described protocol with a minor adjustment.^32^ Rats from the Mod and taVNS groups were subjected to a range of stressors, encompassing various stimuli as follows: wet bedding for 24 h (200 mL of water per cage), day and night reversal for 24 h, cold swimming for 5 min (at 4°C), food deprivation for 24 h, water deprivation for 24 h, restrained for 24 h, tail nip for 1 min (1 cm from the end of the tail), and inversion of the light/dark cycle for 24 h. The rats were subjected to one of these stimulations randomly each day, with no consecutive application of the same stressor on 2 consecutive days in order to prevent the rats from predicting its occurrence. The control group remained undisturbed, except for necessary procedures such as routine cage cleaning.

### 2.3 Transcutaneous auricular vagus nerve stimulation protocol

After depressive‐ and anxiety‐like behaviors were evaluated on PND 54, the taVNS group received three consecutive weeks of therapy intervention. The rats were anesthetized using nasal cone inhalation with 1%–2% induction and 1.5% maintenance of isoflurane (RWD Life Science Co., Ltd., Shenzhen, China). Subsequently, they were connected to an electroacupuncture apparatus (HANS‐200A, Nanjing Jisheng Medical Technology Co., Ltd.) for taVNS. During the intervention, a pair of opposing magnetic electrodes and a custom‐made metal ear splint (2 × 0.5 × 0.05 cm) were placed on the bilateral ear skin of the rats, specifically targeting the auricular concha to establish an electrical circuit (as shown in Figure 1B). The stimulus parameters were as follows: the frequency was set at 2/15 Hz (alternating between 2 and 15 Hz every second), while the intensity was set to 2 mA for a duration of 30 min per day. The taVNS procedure was conducted between 10:00 a.m. and 12:00 noon daily to minimize the impact of biorhythms.

### 2.4 Behavior tests

#### 2.4.1 Open‐field test (OFT)

The OFT test was commonly employed to assess spontaneous locomotor activity and exploratory behavior toward novel environments in rodents. The assessment took place in a square open‐field apparatus, where the test arena consisted of black‐painted walls and floors. The base was divided into equal squares. The central area was defined as a square arena with a diameter of 40 cm in the middle of the apparatus. Each rat was gently introduced into the central arena and observed for a duration of 3 min to record its activity. Parameters assessed were as follows: (1) distance traveled; (2) resting time; (3) ambulatory time; and (4) time in the center.

#### 2.4.2 Elevated plus maze (EPM) test

The EPM test was employed to evaluate anxiety‐like behaviors in rodents. The testing platform consisted of a pair of opened arms, a pair of closed arms, and a central platform connecting the four limbs. At the onset of each session, the rats were positioned within the central square area of the maze, facing one of the open arms, and granted unrestricted freedom to explore. The number of entries and the duration spent in the closed arms during 5 min were meticulously recorded. After each trial, the apparatus was thoroughly cleansed with 75% ethanol solution to eliminate any residual odors.

#### 2.4.3 Forced swim test (FST)

The FST is commonly employed to assess the extent of behavioral despair in rodents. Briefly, rats were acclimated to the experimental environment by being subjected to a 10 min swimming session in water (22–24°C) 1 day prior to the experiment. Then, each rat swam in the same condition in the formal experiment for 5 min while a video camera recorded the immobility time of the rats clearly. The duration of complete immobility was measured during the 3‐min testing period. Immobility was deemed if a rat floated, ceased struggling, or made necessary movements to keep its head above the water.

#### 2.4.4 New object recognition (NOR) test

The NOR test is widely employed for assessing recognition memory in rodents. The NOR protocols comprise three stages: adaptation, training, and testing, with a 24‐h delay between each stage. During the adapting phase, rats were allowed to freely explore the empty test chamber for 10 min. During the training phase, a single rat is introduced to an arena containing two identical cubes positioned in opposite corners, with sufficient space for the rat to explore around them.

During the test session, rats were reintroduced to the arena and presented with one familiar object (a cube) that was previously encountered during training, as well as one unfamiliar object (a cylindrical shape) that had not been encountered before. During the test, three groups of rats were allowed to freely explore the objects and test arenas for 5 min once again. The time spent exploring each object was operationally defined as the duration when the rat positioned its nose within 2 cm of the object. The discrimination index was calculated as follows: [time spent exploring the novel object/total time spent exploring both objects] *100%.

### 2.5 Tissue preparation and sequencing

Male rats were sacrificed under anesthesia by intraperitoneal injection of 1% sodium pentobarbital (50 mg/kg) at postnatal Day 84. The boundaries of the brain tissues selected for this study were determined based on Paxinos and Watson's rat brain atlas. The mPFC, ACC, and HIP were isolated rapidly on ice, placed in the RNAlater solution (Ambion, cat. # AM7020, TX, USA), frozen, and stored at −80°C for further analysis. The TRIzol Reagent (Invitrogen, cat. # 15596‐018) was utilized for the analysis of frozen rat tissues, and subsequent isolation of total RNA was performed. The quality and quantity of RNA were assessed using an Agilent Bioanalyzer (Agilent Technologies, CA, USA).

Prior to mRNA analysis, sample pooling was performed every five samples within the same group. In brief, polyadenylated mRNA was isolated from total RNA using oligo (dT) beads and subsequently fragmented into smaller fragments through the application of divalent cations and thermal treatment. Then, reverse transcription process is carried out by random primers to synthesize the cDNA. The second strand of cDNA was synthesized by adding second‐strand marking buffer and second‐strand/end‐repair enzyme mix. After repairing the terminal, adding base A and sequencing connector, the target fragment was recovered through magnetic bead screening. Subsequently, PCR amplification was conducted to complete the entire library preparation process.

After completion of library construction, Qubit3.0 was utilized for preliminary quantification and the library was subsequently diluted to a concentration of 1 ng/μL. The insert size of the library was then determined using Agilent 2100. Additionally, to accurately quantify the effective concentration of the library and ensure its quality, a Bio‐RAD KIT iQ SYBR GRN was employed in conjunction with Q‐PCR. Finally, qualified libraries were subjected to sequencing on the Illumina PE150 platform.

### 2.6 Statistical analysis

All the experimental data were expressed as the mean ± standard deviation unless otherwise indicated. Data were analyzed by Social Science Statistical Software (SPSS) 26 (IBM, Armonk) and GraphPad Prism 8.0 (GraphPad Software Inc.).

#### 2.6.1 Behavioral tests

The analysis of body masses and behavioral tests among different groups was conducted by one‐way analysis of variance test (ANOVA) followed by post hoc comparisons. Non‐parameter tests were performed by Kruskal–Wallis (K‐W). A probability level of *p*‐value <0.05 was considered statistically significant.

#### 2.6.2 RNA‐Seq data analysis

The raw data obtained through sequencing, in the FASTQ format, were subjected to stringent quality control measures to ensure reliability and accuracy. This involved filtering out low‐quality reads, adapter‐containing reads, and ploy‐N‐containing reads. Furthermore, the clean data were analyzed using Illumina Casava 1.8 software to determine Q30 base rate and GC percent, which served as crucial parameters for subsequent analyses. Fragments per kilobase of transcript per million (FPKM) were calculated using the Limma R package (1.18.0). The threshold criterion for differentially expressed genes (DEGs) has been set at fold change >2 and significance *p*‐value <0.05.

The enrichment analysis of differentially expressed genes (DEGs) in the Gene Ontology (GO) and Kyoto Encyclopedia of Genes and Genomes (KEGG) databases was performed using the GOseq R package and KOBAS software. GO analysis with false discovery rate (FDR)‐adjusted *p*‐values <0.05 was considered significantly enriched by DEGs. For KEGG analysis, terms with an adjusted *p*‐values of 0.05 were considered enriched. The gene expression data were subjected to gene set enrichment analysis (GSEA) using R 3.3.1 software (www.r‐project.org) and GSEA software. For GSEA analysis, a false discovery rate (FDR) < 0.25 was considered statistically significant. The network was constructed by retrieving a curated set of target genes from the database utilizing KOBAS 3.0.^33^ The Venn tool (http://jvenn.toulouse.inra.fr/app/example.html) was utilized to generate Venn diagrams illustrating the DEGs.

## 3 RESULTS

### 3.1 Behavioral results after modeling but prior to the taVNS

As shown in Figure 1C, there was no significant difference in body weights among groups at baseline (PND 28, *p* > 0.05). After the CUMS molding (PND 49), the body weights in the Mod and taVNS groups were significantly decreased compared to the Con group (Figure 1C, *p* < 0.01). In OFT, after modeling (PND 49), the distance traveled, ambulatory time, and time in center decreased and resting time increased in the Mod and taVNS groups compared to the Con group (Figure 1D–H, *p* < 0.01). In FST, the immobility times in both the Mod and taVNS groups were considerably longer than that in the Con group (Figure 1I, *p* < 0.01), so are the time in closed arms and the closed‐arm entries as observed in the EPM test (Figure 1J–L).

### 3.2 Behavioral results after taVNS intervention

**FIGURE 2:**
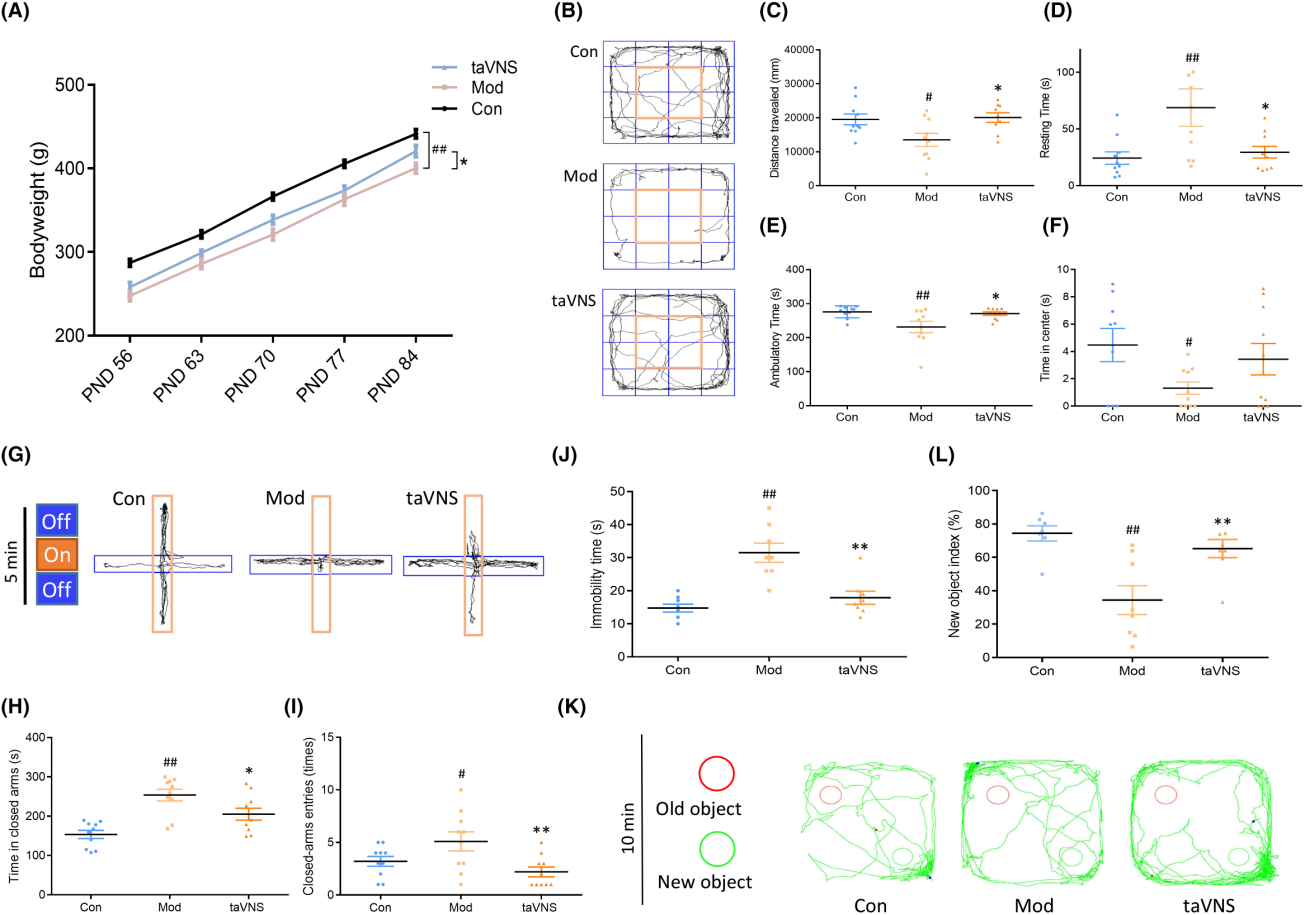
Behavioral tests after taVNS. (A) The effect of taVNS on the body weight at different time points. (B) Movement track of rats in three groups in OFT. (C) Comparison of the distance traveled in OFT. (D) Comparison of the resting time in OFT. (E) Comparison of the ambulatory time in OFT. (F) Comparison of the time in center in OFT. (G) Movement track of rats in three groups in EPM test. (H) Comparison of the time in closed arms in EPM test. (I) Comparison of the closed arms entries in EPM test. (J) The effect of taVNS in FST. (K) Movement track of rats in three groups in NOR test. (L) Comparison of new object index in NOR test. Body weight, OFT, and EPM data were expressed as the mean ± SD (*n* = 10 per group); FST and NOR data were expressed as the mean ± SD (*n* = 8 per group); #*p* < 0.05 versus the control group ##*p* < 0.01 versus the control group; *p < 0.05 versus the model group, **p < 0.01 versus the model group.

As shown in Figure 2A, the weight in Mod group was significantly decreased compared with Con group (PND 84, *p* < 0.01). After 21 days of taVNS intervention, the body weight in CUMS‐exposed rats significantly increased (PND 84, *p* < 0.05, Figure 2A). Meanwhile, similar to the earlier results, the distance traveled (*p* < 0.05), ambulatory time (*p* < 0.01), and time in center (*p* < 0.05) of rats in Mod group were still shorter compared with the Con group, while the resting time was longer (*p* < 0.01) (Figure 2B–F). Additionally, the time in closed arms (*p* < 0.01), closed arms entries (*p* < 0.05), and the immobility time (*p* < 0.01) in the Mod group were longer than that in Con group (Figure 2G–J). As observed in NOR test, the new object index of rats in the Mod groups decreased significantly compared with the Con group (*p* < 0.01, Figure 2K–L). However, after taVNS intervention, most of aforementioned indicators were changed. Specifically, after 21 days of taVNS intervention, the ascending trend of the body weight was further elevated and the final weight at PND 84 approached that in the Con group. Furthermore, the large deviations of all other indicators brought by the CUMS molding were well rectified by the taVNS, which makes the Con and taVNS groups almost at the same level.

### 3.3 taVNS altered gene expression in HIP

The transcriptomic profiles in the HIP, mPFC, and ACC of rats were characterized after taVNS intervention, with sample pooling conducted every five samples within each group prior to mRNA sequencing. The library yielded a total of clean reads after data filtering, with a Q30 quality score ≥91.04% for each sample (Table S1). The mapping rate of clean reads to the rat reference genome (Rnor_6.0) ranged from 95.3% to 98.14%, and the majority (86.07%–92.05%) of them were uniquely mapped (Table S2). These findings indicate that the RNA‐seq data obtained are reliable and suitable for further analysis.

**FIGURE 3:**
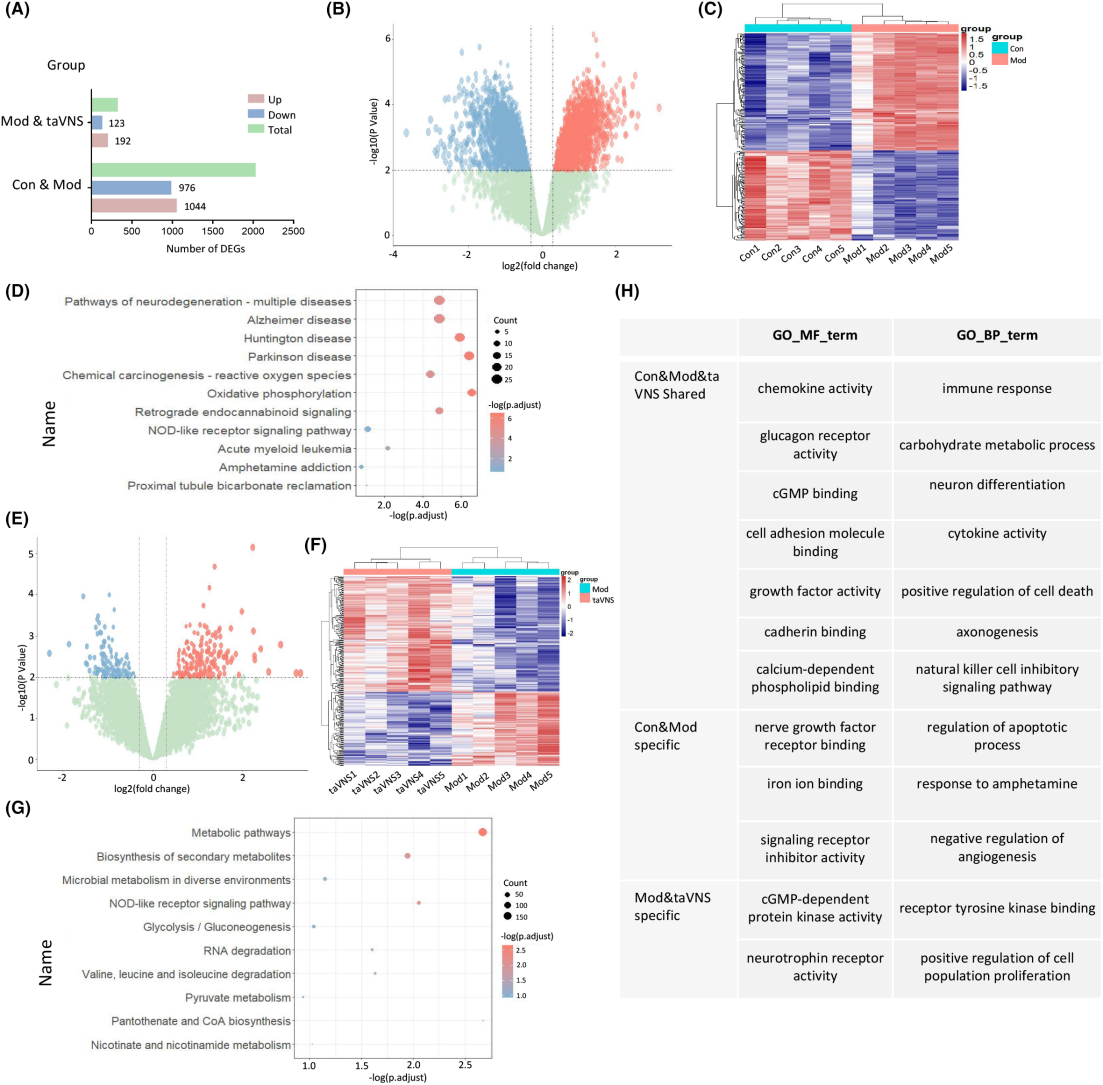
taVNS altered gene expression in HIP. (A) Comparison of DEG numbers in the HIP of three groups. (B) Transcriptomic alterations in the HIP of rats between the Con and Mod groups. Log2 (fold change) plotted relative to ‐log10 (*p*‐value) by volcano plot. Blue and red squares represent down‐ and up‐regulated DEGs that reach significance, log2 FC, and *p* < 0.01 cutoffs, respectively. (C) Heatmap showing the DEGs detected in the comparison between the Con versus Mod groups. Hierarchical clustering of transcriptional profiles in the HIP of the Con versus Mod groups. The color key represents FPKM‐normalized log2‐transformed counts, with each row representing a gene (control group in blue and model group in pink). Subjects within the same groups exhibit clustering patterns. (D) KEGG enrichment analysis of DEGs. The representation of each point corresponds to a specific KEGG term, with the size indicating the number of enriched genes and the color intensity reflecting the *p*.adjust value. (E) Transcriptomic alterations in the HIP of rats between the Mod and taVNS groups. Log2 (fold change) plotted relative to ‐log10 (*p*‐value) by volcano plot. Blue and red squares represent down‐ and up‐regulated DEGs that reach significance, log2 FC, and *p* < 0.01 cutoffs, respectively. (F) Heatmap showing the DEGs detected in the comparison between the Mod versus taVNS groups. Hierarchical clustering of transcriptional profiles in the HIP of the Mod versus taVNS groups. The color key represents FPKM‐normalized log2‐transformed counts, with each row representing a gene (model group in blue and taVNS group in pink). Subjects within the same groups exhibit clustering patterns. Subjects within groups cluster together. (G) KEGG enrichment analysis of DEGs. The representation of each point corresponds to a specific KEGG term, with the size indicating the number of enriched genes and the color intensity reflecting the *p*.adjust value. (H) GO enrichment analysis. GO_MF_term, molecular function of GO analysis. GO_BP_term, biological process of GO analysis.

A total of 2020 DEGs were identified in the HIP, including 1044 up‐regulated and 976 down‐regulated genes in the Mod group compared with the Con group (Figure 3A–C). Compared to the Con group, the Mod group exhibited significantly enriched KEGG pathways, particularly in relation to “Oxidative phosphorylation” (OXPHOS) and neurodegenerative disorders including Alzheimer's disease (AD), Huntington's disease (HD), and Parkinson's disease (PD) (Figure 3D).

There were 315 DEGs (192 up‐regulated and 123 down‐regulated) in the taVNS group compared with the Mod group (Figure 3A,E,F). Those DEGs were mainly related to metabolic pathways, such as “Biosynthesis of secondary metabolites,” “Microbial metabolism in diverse environments,” and “Nicotinate and nicotinamide metabolism” (Figure 3G).

Furthermore, GO enrichment analysis suggested that DEGs between Con and Mod groups were enriched in GO Molecular Function (MF) term: “iron ion binding,” “GO MF term: nerve growth factor receptor binding,” “GO Biological Process (BP) term: regulation of apoptotic process,” “GO BP term: response to amphetamine,” etc. In addition to this, DEGs between Mod and taVNS groups were enriched in GO MF term: “cGMP‐dependent protein kinase activity,” “GO MF term: neurotrophin receptor activity,” “GO BP term: receptor tyrosine kinase binding,” “GO BP term: positive regulation of cell population proliferation,” etc. (Figure 3H).

### 3.4 taVNS repressed inflammatory response and activated glycolysis in HIP

**FIGURE 4:**
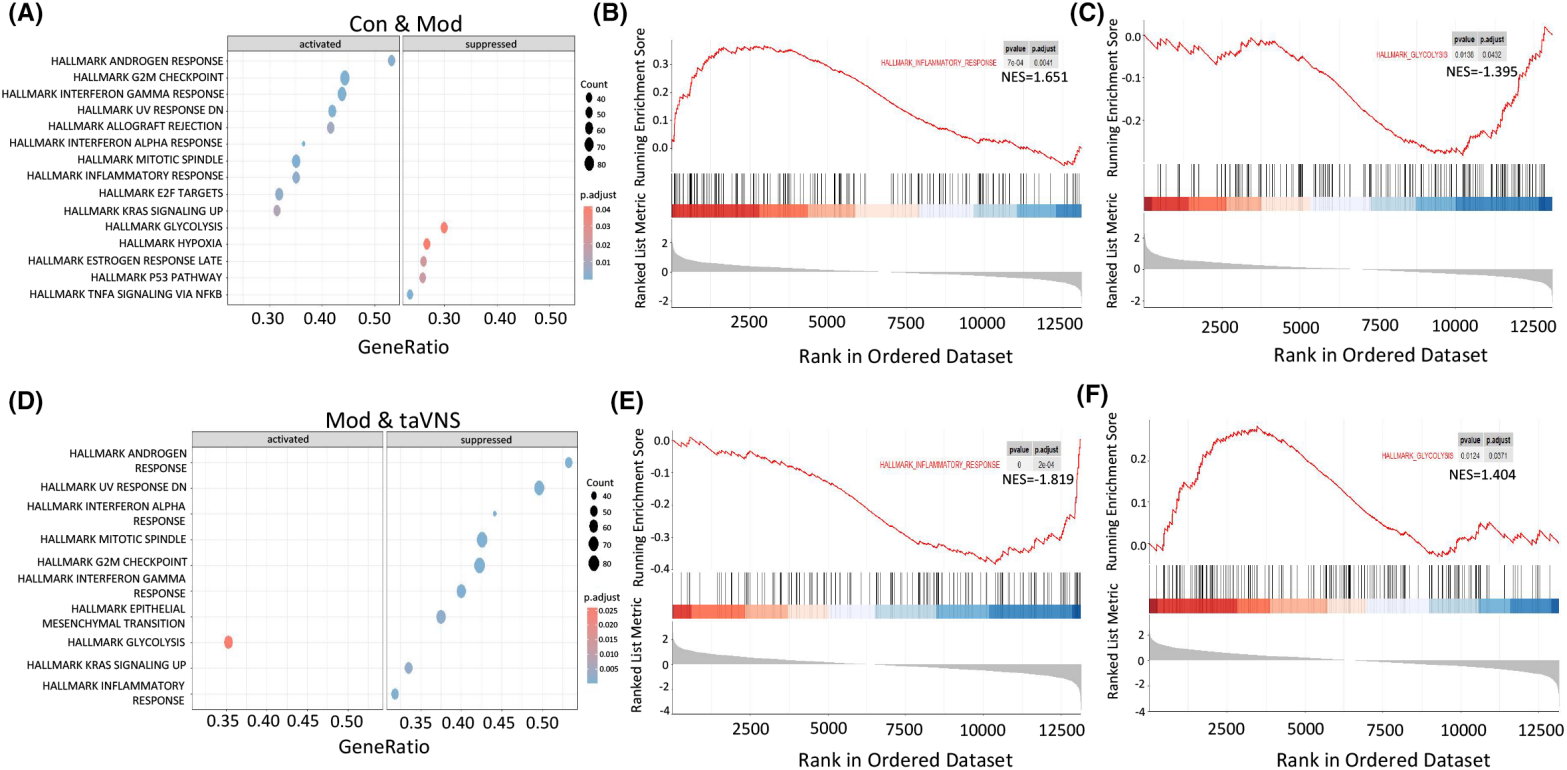
GSEA analysis revealed the effects of taVNS on different pathways in HIP. (A) Comparison between the Con and Mod groups. The representation of each point corresponds to a specific KEGG term, with the size indicating the number of enriched genes and the color intensity reflecting the *p*.adjust value. (B) The GSEA analysis revealed that inflammatory response‐associated genes were elevated. (C) The GSEA analysis revealed that glycolysis‐associated genes were suppressed in the Mod group compared with the Con group. (D) Comparison between the Mod and the taVNS group. The representation of each point corresponds to a specific KEGG term, with the size indicating the number of enriched genes and the color intensity reflecting the p.adjust value. (E) The GSEA analysis revealed that inflammatory response‐associated genes were suppressed. (F) The GSEA analysis revealed that glycolysis‐associated genes were elevated in taVNS group compared with Mod group.

Then, we performed GSEA analysis to evaluate the effects of taVNS on different pathways in HIP. The results suggested that “INFLAMMATORY RESPONSE” activated and “GLYCOLYSIS PATHWAY” were suppressed in the Mod group compared with the Con group (Figure 4A). Those two pathways were significantly enriched, with NES values of 1.651 and −1.395, respectively (Figure 4B,C). Notably, taVNS intervention reversed the main signaling that changed significantly in the Mod group, with NES values of −1.819 and 1.404 for the “INFLAMMATORY RESPONSE” and “GLYCOLYSIS PATHWAY,” respectively (Figure 4D–F).

**FIGURE 5:**
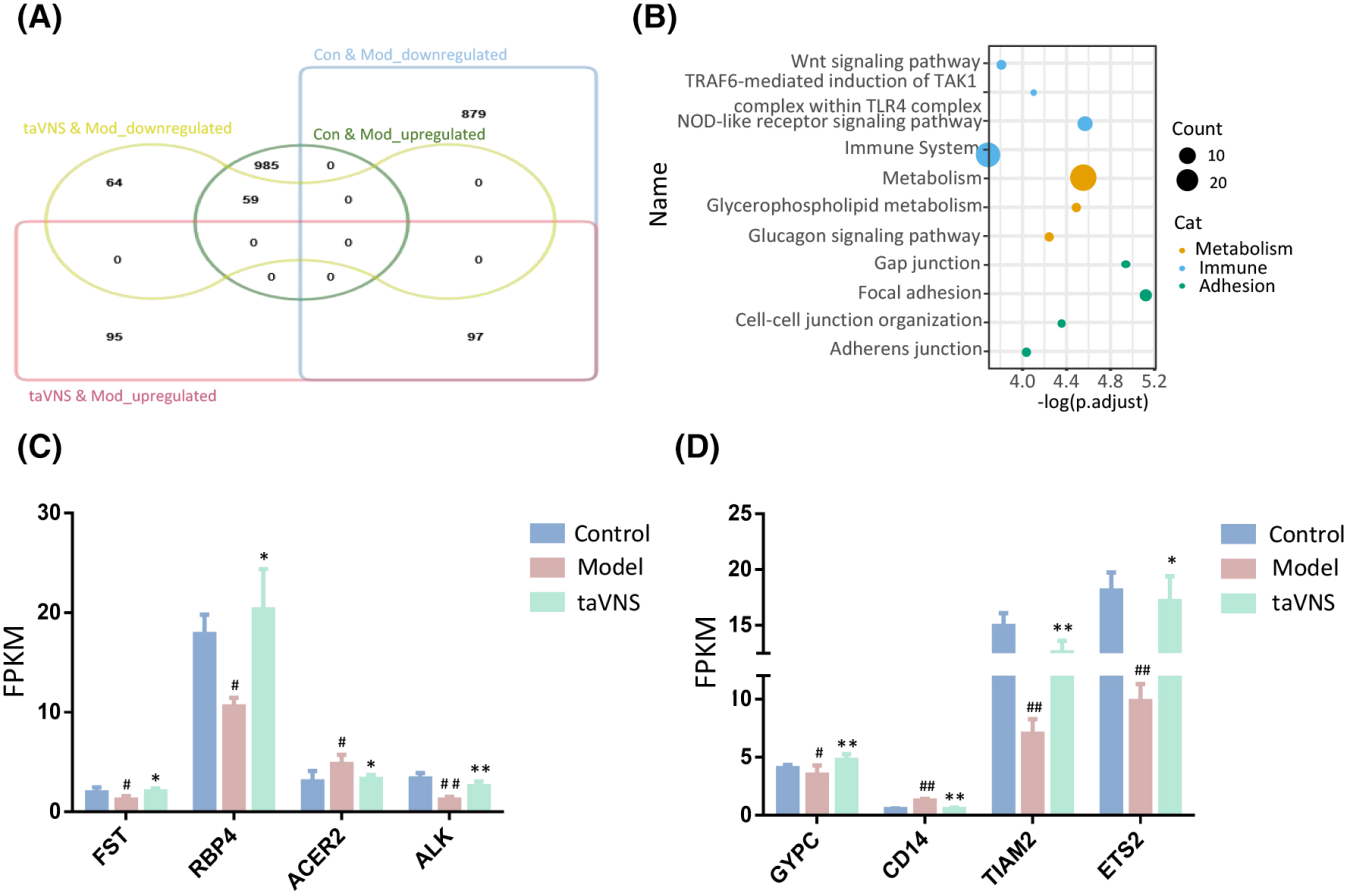
taVNS rescued the abnormal expression of metabolism‐ and immune‐associated DEGs in HIP. (A) The Venn diagram showed the overlapped DEGs among different comparisons. (B) KEGG analysis of overlapped DEGs. The representation of each point corresponds to a specific KEGG term, with the size indicating the number of enriched genes and the color intensity reflecting the p.adjust value. Yellow point represents metabolism; blue point indicates immune; and green point indicates adhesion. (C) Expression of metabolism‐associated DEGs. (D) Expression of immune‐associated DEGs. Data were expressed as the mean ± SD (*n* = 5 per group); #*p* < 0.05 versus the control group, ##*p* < 0.01 versus the control group; *p < 0.05 versus the model group, **p < 0.01 versus the model group.

Among all 976 down‐regulated genes in the Mod group, there were 97 genes up‐regulated after taVNS intervention. Correspondingly, among all 1044 up‐regulated in the Mod group, there were 59 genes down‐regulated in the taVNS group (Figure 5A). In the KOBAS analysis, the most enriched KEGG pathways of those overlapped genes were divided into three categories, that is, “Metabolism,” “Immune regulation,” and “Cell adhesion.” For the “Metabolism” category, “Glycerophospholipid metabolism” and “Glucagon signaling pathway” were highly enriched. For the immune regulation category, “Wnt signaling pathway,” “TRAF6‐mediated induction of TAK1 complex within TLR4 complex,” and NOD‐like receptor signaling pathway” were significantly enriched (Figure 5B). The expression of representative immune‐ and metabolism‐related DEGs in different groups was shown in bar plots (Figure 5C,D). Notedly, the expression of those DEGs was reversed by taVNS (Figure 5C,D).

### 3.5 taVNS treatment altered the metabolism and immune response in mPFC

**FIGURE 6:**
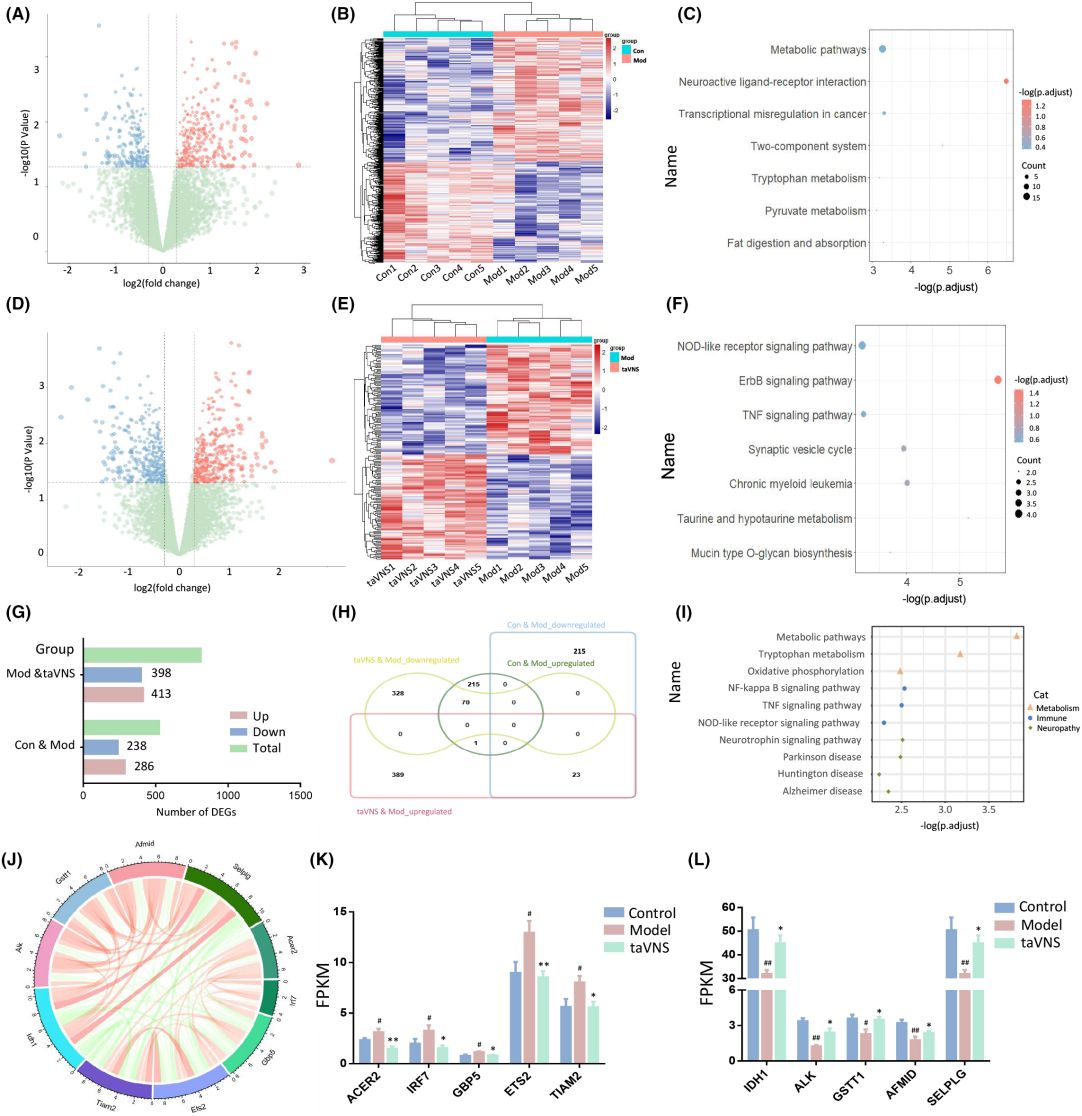
taVNS altered gene expression in mPFC. (A) Transcriptomic alterations in the mPFC between the Con and Mod groups. (B) Heatmap showing the DEGs detected in the comparison between the Con versus Mod groups. (C) KEGG enrichment analysis of DEGs. (D) Transcriptomic alterations in the mPFC of rats between the Mod and taVNS groups. (E) Heatmap showing the DEGs detected in the comparison between the Mod versus taVNS groups. (F) KEGG enrichment analysis of DEGs. (G) Comparison of DEG numbers in the mPFC of three groups. (H) The Venn diagram showed the overlapped DEGs among different comparisons. (I) KEGG analysis of overlapped DEGs. (J) Circos plot displaying interconnectivity among genes altered by taVNS. (K) Expression of Immune‐associated DEGs. (L) Expression of metabolism‐associated DEGs. Data were expressed as the mean ± SD (*n* = 5 per group); #*p* < 0.05 versus the control group, ##*p* < 0.01 versus the control group; *p < 0.05 versus the model group, **p < 0.01 versus the model group.

A total of 524 DEGs were identified in the mPFC, including 286 up‐regulated and 238 down‐regulated genes in the Mod group compared with the Con group (Figure 6A,B). Among them, the most enriched KEGG pathways were “Metabolic pathways,” “Neuroactive ligand‐receptor interaction,” etc. (Figure 6C). There were 811 DEGs (413 up‐regulated and 398 down‐regulated) in the taVNS group compared with the Mod group (Figure 6D,E), which were mainly related to NOD‐like receptor/ErbB/TNF signaling pathways (Figure 6F).

Among all 238 down‐regulated genes in the Mod group, there were 23 genes up‐regulated after taVNS intervention. However, among all 286 up‐regulated genes in the Mod group, 70 of them were down‐regulated in the taVNS group (Figure 6G,H). In the KOBAS analysis, the most enriched KEGG pathways of those overlapped genes were mainly divided into three categories: “Metabolism,” “Immune” and “Neuropathy.” For metabolism category, “Tryptophan metabolism” and “Oxidative phosphorylation” were highly enriched. For the immune regulation category, “NF‐kappa B signaling pathway,” “TNF signaling pathway,” and “NOD‐like receptor signaling pathway” were significantly enriched (Figure 6I). The expression of representative Immune‐ and Metabolism‐related DEGs in different groups is shown in Figure 6J–L. Notedly, all of those genes were significantly reversed by taVNS.

### 3.6 taVNS treatment altered the metabolism and immune response in ACC

**FIGURE 7:**
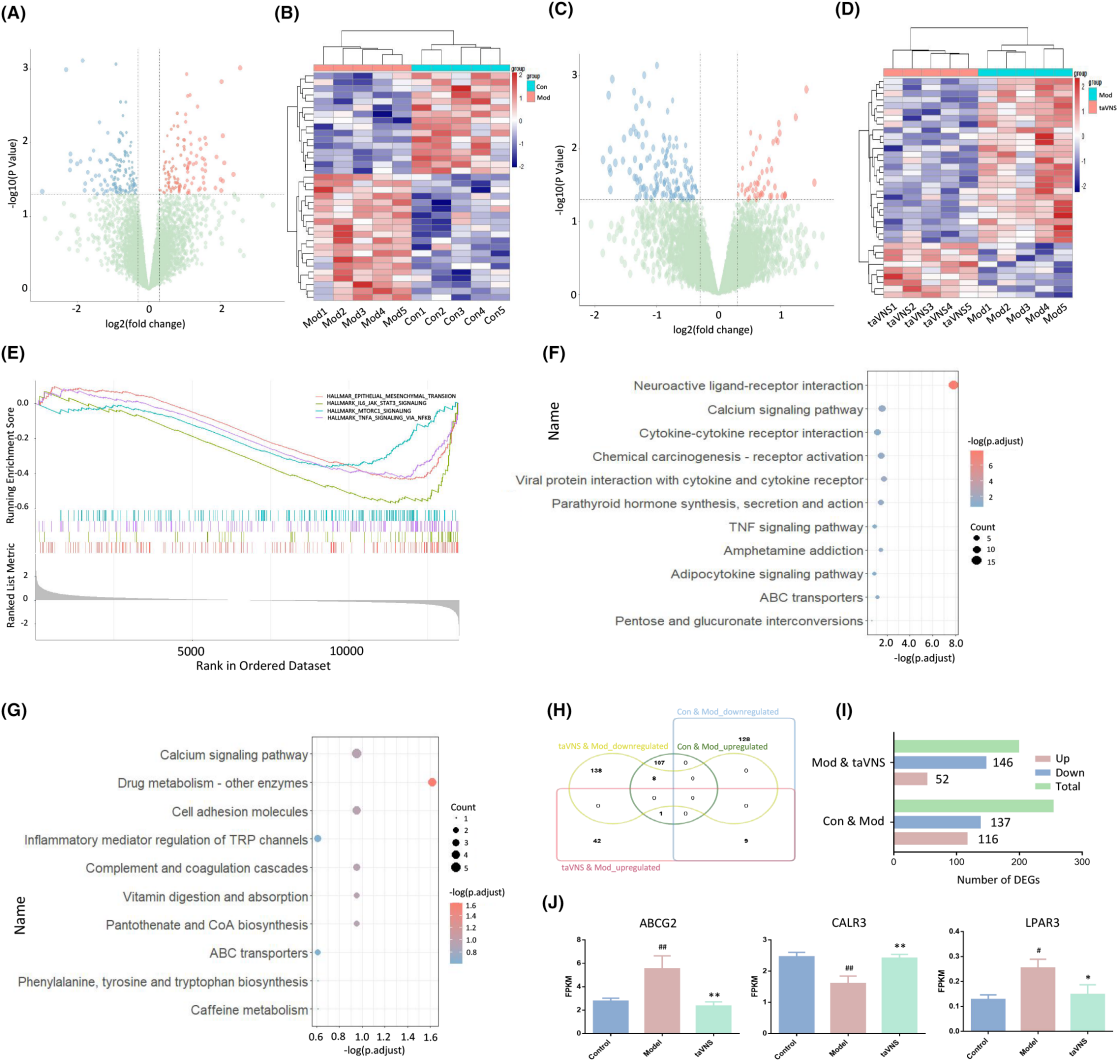
taVNS altered gene expression in ACC. (A) Transcriptomic alterations in the ACC of rats between the Con and Mod groups. (B) Heatmap showing the DEGs detected in the comparison between the Con versus Mod groups. (C) Transcriptomic alterations in the ACC of rats between Mod and taVNS groups. (D) Heatmap showing the DEGs detected in the comparison between the Mod versus taVNS groups. (E) GSEA analysis multiple signal pathways were altered between Con and Mod groups. (F) KEGG analysis of DEGs between Con and Mod groups. (G) KEGG pathway analysis of DEGs between Mod and taVNS groups. (H) The Venn diagram showed the overlapped DEGs among different comparisons. (I) Comparison of DEG numbers in the PFC of three groups. (J) Expression of calcium‐signaling pathway and ABC transporters associated DEGs. Data were expressed as the mean ± SD (*n* = 5 per group); #*p* < 0.05 versus the control group, ##*p* < 0.01 versus the control group; *p < 0.05 versus the model group, **p < 0.01 versus the model group.

A total of 253 DEGs were identified in the ACC, including 116 up‐regulated and 137 down‐regulated genes in the Mod group compared with the Con group (Figure 7A,B). There were 198 DEGs (52 up‐regulated and 146 down‐regulated) in the taVNS group compared with Mod group (Figure 7C,D). GSEA analysis showed that “EPITHELIAL MESENCHYMAL TRANSITION” (NES = −1.800, *p*.adjust <0.001), “IL6 JAK_STAT3_SIGNALING” (NES = −1.976, *p*.adjust < 0.001), “MTORC1 SIGNALING” (NES = −1.522, *p*.adjust < 0.005), and “TNFA SIGNALING VIA NFKB” (NES = −1.743, *p*.adjust < 0.001) were significantly suppressed in the Mod group compared with the Con group (as shown in Figure 7E, all *p*.adjust values were above 0.01 and FDRs were above 0.25). Yet, no GSEA pathways were significantly enriched in the taVNS group, suggesting that the effect brought by taVNS is mild and dispersed. In the KEGG enrichment analysis, between Con and Mod groups, “Neuroactive ligand‐receptor interaction,” “Calcium signaling pathway,” “Cytokine‐cytokine receptor interaction,” “TNF signaling pathway,” and “Amphetamine addiction” were the top‐ranked terms (Figure 7F). Remarkably, “Calcium signaling pathway” and “ABC transporters” were also significantly enriched in taVNS group compared with Mod group (Figure 7G).

Among 137 down‐regulated genes in the Mod group, there were nine genes up‐regulated in the taVNS group. Additionally, there were eight genes up‐regulated in the Mod group and down‐regulated under taVNS (Figure 7H,I). However, for the above 17 genes, no significantly enriched KEGG term was obtained by GSEA. The expression of representative DEGs associated with calcium signaling and ABC transporters in different groups was shown in bar plots, and all those genes were significantly reversed by taVNS (Figure 7J).

## 4 DISCUSSION

### 4.1 Effects of taVNS on CUMS‐induced depressive and anxiety‐like behaviors

The heightened neuroplasticity observed during adolescence renders the brain particularly susceptible to environmental stress, thereby disrupting developmental processes in brain regions responsible for emotional and cognitive processing, ultimately leading to the manifestation of depressive and anxiety‐like behaviors.^11^, ^34^ CUMS is a classical model that mimics the role of naturalistic socio‐environmental stressors‐induced depression, and CUMS in the adolescent period can lead to persistent maladaptive behaviors.^35^, ^36^, ^37^ A previous study reported that male rats induced by adolescent stresses contributed to depression susceptibility in adulthood,^38^ and similar results were seen in monkey models.^39^

In this study, we found that adolescent (PND 28–49) rats exposed to CUMS induced depressive and anxiety‐like behaviors in the short‐term (late‐adolescent, PND 50), which still persisted in adulthood (PND 76). These results suggest that ELA stability could cause long‐lasting behavioral disorders.^40^, ^41^ Nonetheless, adult depression was quite distinct from adolescent depression, and pharmacological interventions for major depressive disorder (MDD) in the adolescent population were found unsatisfactory.^42^ Hence, in this work, we proposed taVNS as a potentially complementary therapy for depression and demonstrated for the first time that 3 weeks of taVNS treatment could reverse depressive‐ and anxiety‐like behaviors in both adolescence and adulthood.

### 4.2 The regulatory effects of taVNS on different brain regions

The etiology of adolescent depression involves various developmental stages, diverse brain regions, and different types of brain cells, making it a complex and heterogeneous psychiatric disorder.^43^, ^44^, ^45^, ^46^ The activation and dysregulation engulfment capacity of microglia in the ACC have frequently been identified as triggering factors for aberrant immune responses in adolescent depression.^47^ Additionally, stresses during adolescence induce depressive behaviors, abnormal neuronal development, and alter the excitatory–inhibitory balance in the PFC and HIP.^48^, ^49^ The development of non‐pharmacological therapies that improve the functioning of multiple brain regions or cell types holds greater clinical significance. Here, we revealed the effects of taVNS on three depression‐related brain regions, which basically responded differently to the stimulation in terms of metabolism. For the mPFC region, we also observed a significant enrichment of DEGs associated with PD, HD, and AD. This suggested that depression may share comorbidity mechanisms with these neurodegenerative diseases, and taVNS may also have a general therapeutic effect on different nervous system diseases. As to the ACC region dealing with stress and emotional outcomes, its calcium signaling was significantly altered by taVNS, suggesting a critical role of taVNS in regulating depolarization signaling and synaptic activity. For the HIP region, taVNS mainly regulated genes involved in cell adhesion (gap junction, focal adhesion, cell–cell junction organization, etc.). Previously, the focal adhesion‐PI3K‐Akt–mTOR‐signaling pathways were regarded as potential comorbidity mechanisms shared by schizophrenia (SZ), bipolar disorder (BD), and MDD.^50^ Here, we reveal for the first time that cell adhesion in the HIP could be a therapeutic target to improve depressive behavior. In conclusion, we provide a novel perspective for the “multi‐target, multi‐pathway” effects of taVNS on depression and highlight taVNS as a potential non‐pharmacotherapy for nervous system diseases.

### 4.3 taVNS may improve depressive and anxiety‐like behaviors by regulating HIP

Although the neural circuits underlying depression pathology remain unclear, the diverse symptoms of depression suggest that multiple brain regions may be implicated.^51^ According to previous reports, the HIP, ACC, and PFC are the most affected brain regions after stresses.^52^ Here, we investigated the impacts of taVNS on those three brain regions in rats subjected to CUMS. Our findings revealed that taVNS exerted effects in a cerebral region‐specific manner. Most previous reports focused on the regulation of immunity by taVNS^27^; here, we proposed that the regulation of disordered metabolism across different brain regions is also an important feature of taVNS. Notably, taVNS treatment almost completely recovered the signaling pathways altered by CUMS modeling, particularly in HIP, suggesting that taVNS may improve depressive behaviors and cognitive impairments by regulating gene expression in HIP.

### 4.4 taVNS can restore the suppressed glycolysis in HIP caused by CUMS

Depression is often accompanied by disrupted energy metabolism.^53^, ^54^, ^55^ In the present study, metabolism alterations were identified in all three brain regions of CUMS rats. Previous studies have demonstrated the inhibition of the tricarboxylic acid (TCA) cycle in mice exposed to chronic social defeat stress (CSDS) and patients experiencing first‐episode depression.^56^ Also, the depression‐like behavior was associated with significant alternations of both glycolysis and TCA cycle in different brain regions.^57^, ^58^ Glucose served as the primary energy source for the developmental brain, facilitating proper neurotransmitter synthesis and function, particularly with regard to glutamate and γ‐aminobutyric acid (GABA), as well as appropriate nicotinamide adenine dinucleotide phosphate oxidase (NADPH) levels. The olfactory bulbectomized rat models of depression demonstrated a reduction in overall cerebral glucose utilization,^59^ while rats exposed to CUMS exhibited insulin resistance specifically within the arcuate nucleus of the hypothalamus.^60^ Hence, glucose metabolism dysfunction was strongly involved in the pathogenesis of depression.^55^ Here, we demonstrated for the first time that 3 weeks of taVNS effectively ameliorated CUMS‐induced glycolysis decline in the HIP.

### 4.5 taVNS can suppress the activated immune response in HIP caused by CUMS

A dysregulated immune response was widely identified in patients with MDD.^61^ This phenomenon was also observed in both rodent and non‐human primate models.^62^, ^63^, ^64^ A series of previous studies have consistently demonstrated that early life stresses, mainly maternal separation, can reliably induce depression‐like behaviors in experimental animals and exhibit significant neuroinflammatory characteristics in the HIP.^65^, ^66^, ^67^ Given the anti‐inflammatory properties of numerous antidepressant medications, neuroimmune mechanisms are now considered pivotal in the pathogenesis of depressive symptoms.^68^, ^69^ Here, we found that immune response was significantly enriched in HIP and mPFC of CUMS‐exposed rats, and taVNS could significantly rescue this change. Particularly, the GSEA results suggested that the immune response disorder caused by CUMS may be reversed by taVNS mainly via the Wnt/ NOD‐like receptor signaling pathway. Previously, we found that CUMS rats exhibited neuroinflammatory features in the HIP, and taVNS ameliorated depressive behavior via the α7 nicotinic acetylcholine receptors (α7nAchR)/NF‐κB signaling pathway.^70^ Here, we further elucidated and expanded upon the signaling pathway underlying the anti‐inflammatory effects of taVNS that the Wnt/ NOD‐like receptor signaling pathway in HIP was also deeply involved in the antidepressant effects of taVNS.

## 5 LIMITATIONS

A few limits were placed on the current investigation: (1) While CUMS stimulation in rats is commonly considered a prototypical animal model for depression, it fails to accurately capture the intricate characteristics of depressed human individuals. To gain deeper insights into the pathways associated with antidepression, additional animal models and clinical investigations are imperative. (2) Only male rats were used in the current study to prevent hormone fluctuations in female rats from affecting neuron structure, neural reward function, and motivation, which were also associated with mental health conditions like depression. Considering the necessity of assessing potential sex bias effects, the current investigation should also be expanded to female rats. (3) We have provided strong evidence that taVNS reduced behavioral indications of depression in rats and had antidepressant effects by preventing inflammatory responses and activating glycolysis in HIP. However, the dysregulated gene expression still needs to be verified, and further molecular biology investigation and proof of the gene functions are required to confirm the hypothesis.

## 6 CONCLUSION

In conclusion, our study has revealed the protective effects of taVNS intervention on depressive‐ and anxiety‐like behaviors in adulthood using an adolescent rat model of stress‐induced depression. This provides a theoretical basis for understanding the antidepressant mechanism. To be specific, the adolescent CUMS rats exhibited depressive‐ and anxiety‐like behaviors, such as reduced locomotor activity, increased anxiety and desperate behavior, impaired learning, and memory ability. We proved that the taVNS intervention in the early stage not only ameliorated the depression in adolescence but in the adulthood period as well. Furthermore, we found that exposure to CUMS affected gene expression in the HIP, mPFC, and ACC, suggesting adolescent stress led to widespread pathological changes in these brain regions. Compared with ACC and mPFC, the effects of taVNS on the HIP were more clearly delineated, indicating the involvement of suppressed inflammatory response and activated glycolysis.

## AUTHOR CONTRIBUTIONS

Lan Sun, Shixiang Ma, and Qianwen Wei performed experiments and acquired data. Lan Sun designed the experiments and wrote the first draft of the manuscript. Xiangji Li and Yun Yu provided experimental help. Li Min and Peijing Rong planned the overall direction of the experiment. Li Min and Peijing Rong supervised the experiment and reviewed the article. All authors have read and approved the final manuscript.

## FUNDING INFORMATION

This study was supported by funding from the National Natural Science Foundation of China (82304914) and the National Key Research and Development Program of China (2022YFC3500500).

## CONFLICT OF INTEREST STATEMENT

The authors declared no potential conflicts of interest with respect to the research, authorship, and/or publication of this article.

## Supporting information


Table S1

## Data Availability

The datasets utilized and/or analyzed in this study can be obtained from the corresponding author upon a reasonable request.
