# Supplementary material for: Transcutaneous auricular vagus nerve stimulation ameliorates adolescent depressive‐ and anxiety‐like behaviors via hippocampus glycolysis and inflammation response
Source: CNS Neurosci Ther. 2024 Feb 15;30(2):e14614. doi: 10.1111/cns.14614 (PMC10867795; doi:10.1111/cns.14614)
Supplement: Supplementary file 1 — Table S1 [file CNS-30-e14614-s001.docx]

**Supplementary Table S1.** Summary of the data quality from RNA-seq

| Sample name | Raw Reads | Raw Bases(G) | Clean Reads | Clean Reads Rate (%) | Clean Bases(G) | Raw Q30 Bases Rate (%) | Clean Q30 Bases Rate (%) | Raw GC percent (%) | Clean GC percent  (%) |
| --- | --- | --- | --- | --- | --- | --- | --- | --- | --- |
| CH1 | 49767510 | 7465126500 | 46835526 | 94.11 | 7025328900 | 95.1 | 94.97 | 46.89 | 46.83 |
| CH2 | 49038752 | 7355812800 | 46321478 | 94.46 | 6948221700 | 95.19 | 95.08 | 46.86 | 46.79 |
| CH3 | 47109706 | 7066455900 | 44966450 | 95.45 | 6744967500 | 95.23 | 95.13 | 47.58 | 47.55 |
| CH4 | 49523768 | 7428565200 | 45975920 | 92.84 | 6896388000 | 95.05 | 94.89 | 48.8 | 48.8 |
| CH5 | 49528556 | 7429283400 | 45890140 | 92.65 | 6883521000 | 95.38 | 95.23 | 49.25 | 49.24 |
| CP1 | 50323854 | 7548578100 | 46037216 | 91.48 | 6905582400 | 94.27 | 94.02 | 50.19 | 50.19 |
| CP2 | 47071302 | 7060695300 | 44911134 | 95.41 | 6736670100 | 92.48 | 92.3 | 52.53 | 52.57 |
| CP3 | 61935346 | 9290301900 | 59068520 | 95.37 | 8860278000 | 93.18 | 93 | 52.21 | 52.25 |
| CP4 | 42543442 | 6381516300 | 40712050 | 95.7 | 6106807500 | 94.64 | 94.53 | 45.56 | 45.48 |
| CP5 | 48179138 | 7226870700 | 46252322 | 96 | 6937848300 | 94.2 | 94.09 | 45.46 | 45.4 |
| CD1 | 44613626 | 6692043900 | 43349334 | 97.17 | 6502400100 | 88.11 | 87.92 | 50.63 | 50.62 |
| CD2 | 49000134 | 7350020100 | 46250172 | 94.39 | 6937525800 | 94.71 | 94.58 | 48.13 | 48.1 |
| CD3 | 50206698 | 7531004700 | 47057736 | 93.73 | 7058660400 | 94.64 | 94.47 | 50.78 | 50.8 |
| CD4 | 60812498 | 9121874700 | 58099274 | 95.54 | 8714891100 | 91.27 | 91.04 | 51.75 | 51.78 |
| CD5 | 64779102 | 9716865300 | 61983816 | 95.68 | 9297572400 | 91.99 | 91.79 | 51.6 | 51.63 |
| MH1 | 43978998 | 6596849700 | 42447224 | 96.52 | 6367083600 | 93.83 | 93.72 | 45.64 | 45.59 |
| MH2 | 40747110 | 6112066500 | 39423926 | 96.75 | 5913588900 | 94.06 | 93.97 | 44.75 | 44.7 |
| MH3 | 43729002 | 6559350300 | 41983338 | 96.01 | 6297500700 | 94.33 | 94.22 | 47 | 46.96 |
| MH4 | 40463770 | 6069565500 | 38667180 | 95.56 | 5800077000 | 92.69 | 92.52 | 46.26 | 46.18 |
| MH5 | 65754896 | 9863234400 | 62771822 | 95.46 | 9415773300 | 91.85 | 91.64 | 51.92 | 51.95 |
| MP1 | 46056880 | 6908532000 | 44459358 | 96.53 | 6668903700 | 93.11 | 93.01 | 43.59 | 43.55 |
| MP2 | 43339726 | 6500958900 | 41627552 | 96.05 | 6244132800 | 94.65 | 94.56 | 46.85 | 46.8 |
| MP3 | 48641436 | 7296215400 | 45769398 | 94.1 | 6865409700 | 94.87 | 94.73 | 47.41 | 47.38 |
| MP4 | 45813934 | 6872090100 | 44091572 | 96.24 | 6613735800 | 93.52 | 93.4 | 43.57 | 43.5 |
| MP5 | 76422578 | 11463386700 | 73542878 | 96.23 | 11031431700 | 92.57 | 92.43 | 44.84 | 44.77 |
| MD1 | 47913826 | 7187073900 | 45688724 | 95.36 | 6853308600 | 92.36 | 92.16 | 51.93 | 51.96 |
| MD2 | 48939216 | 7340882400 | 46990088 | 96.02 | 7048513200 | 92.53 | 92.38 | 52.19 | 52.24 |
| MD3 | 61211048 | 9181657200 | 59557216 | 97.3 | 8933582400 | 92.11 | 92.02 | 44.03 | 43.99 |
| MD4 | 46534978 | 6980246700 | 44340734 | 95.28 | 6651110100 | 92.2 | 92.01 | 52.26 | 52.3 |
| MD5 | 43465898 | 6519884700 | 41368934 | 95.18 | 6205340100 | 92.79 | 92.6 | 51.72 | 51.77 |
| NH1 | 41826924 | 6274038600 | 40145966 | 95.98 | 6021894900 | 94.22 | 94.11 | 46.31 | 46.26 |
| NH2 | 43413962 | 6512094300 | 41309306 | 95.15 | 6196395900 | 94.53 | 94.41 | 46.54 | 46.49 |
| NH3 | 48217414 | 7232612100 | 45261606 | 93.87 | 6789240900 | 92.85 | 92.61 | 49.26 | 49.22 |
| NH4 | 47030542 | 7054581300 | 44053686 | 93.67 | 6608052900 | 94.89 | 94.73 | 48.73 | 48.74 |
| NH5 | 44903858 | 6735578700 | 43276628 | 96.38 | 6491494200 | 94.12 | 94.02 | 48.11 | 48.08 |
| NP1 | 47586216 | 7137932400 | 45430684 | 95.47 | 6814602600 | 94.03 | 93.89 | 47.64 | 47.61 |
| NP2 | 47821416 | 7173212400 | 45901406 | 95.99 | 6885210900 | 92 | 91.82 | 51.9 | 51.94 |
| NP3 | 45886114 | 6882917100 | 44010266 | 95.91 | 6601539900 | 92.31 | 92.14 | 51.55 | 51.59 |
| NP4 | 45148008 | 6772201200 | 42594310 | 94.34 | 6389146500 | 93.14 | 92.94 | 52.57 | 52.61 |
| NP5 | 44855846 | 6728376900 | 43477638 | 96.93 | 6521645700 | 92.49 | 92.36 | 52.13 | 52.15 |
| ND1 | 43639128 | 6545869200 | 41138426 | 94.27 | 6170763900 | 92.38 | 92.11 | 52.05 | 52.1 |
| ND2 | 49432964 | 7414944600 | 47369546 | 95.83 | 7105431900 | 92.9 | 92.73 | 52.03 | 52.07 |
| ND3 | 47846506 | 7176975900 | 45436226 | 94.96 | 6815433900 | 92.64 | 92.44 | 51.64 | 51.68 |
| ND4 | 46318330 | 6947749500 | 43762642 | 94.48 | 6564396300 | 92.74 | 92.52 | 51.95 | 51.99 |
| ND5 | 45100222 | 6765033300 | 43353484 | 96.13 | 6503022600 | 92.84 | 92.69 | 52.23 | 52.26 |

Note: C means Control group, M means Model group, N means taVNS group; H & P & D (HIP & mPFC & ACC).

**Supplementary Table S2.** Overview of the mapped reads

| Sample name | Total Reads | Mapped Reads | Mapping Rate | UnMapped Reads | MultiMap Reads | uniquely mapped | MultiMap Rate |
| --- | --- | --- | --- | --- | --- | --- | --- |
| CH1 | 46835526 | 45915700 | 98.04% | 919826 | 3313386 | 90.97% | 7.07% |
| CH2 | 46321478 | 45440776 | 98.1% | 880702 | 3166012 | 91.27% | 6.83% |
| CH3 | 44966450 | 44128734 | 98.14% | 837716 | 3012377 | 91.44% | 6.7% |
| CH4 | 45975920 | 45062003 | 98.01% | 913917 | 2983476 | 91.52% | 6.49% |
| CH5 | 45890140 | 44961595 | 97.98% | 928545 | 2883345 | 91.7% | 6.28% |
| CP1 | 46037216 | 45025852 | 97.8% | 1011364 | 2937760 | 91.42% | 6.38% |
| CP2 | 44911134 | 43323942 | 96.47% | 1587192 | 2307973 | 91.33% | 5.14% |
| CP3 | 59068520 | 57001329 | 96.5% | 2067191 | 3132138 | 91.2% | 5.3% |
| CP4 | 40712050 | 39827460 | 97.83% | 884590 | 3147656 | 90.1% | 7.73% |
| CP5 | 46252322 | 45310012 | 97.96% | 942310 | 3592914 | 90.19% | 7.77% |
| CD1 | 43349334 | 41725434 | 96.25% | 1623900 | 2338796 | 90.85% | 5.4% |
| CD2 | 46250172 | 45280334 | 97.9% | 969838 | 3172056 | 91.04% | 6.86% |
| CD3 | 47057736 | 46075430 | 97.91% | 982306 | 2758565 | 92.05% | 5.86% |
| CD4 | 58099274 | 55910495 | 96.23% | 2188779 | 3364716 | 90.44% | 5.79% |
| CD5 | 61983816 | 59828309 | 96.52% | 2155507 | 3341871 | 91.13% | 5.39% |
| MH1 | 42447224 | 41465765 | 97.69% | 981459 | 3207349 | 90.13% | 7.56% |
| MH2 | 39423926 | 38572645 | 97.84% | 851281 | 3243602 | 89.61% | 8.23% |
| MH3 | 41983338 | 40977507 | 97.6% | 1005831 | 2848874 | 90.81% | 6.79% |
| MH4 | 38667180 | 37805098 | 97.77% | 862082 | 2729360 | 90.71% | 7.06% |
| MH5 | 62771822 | 60455055 | 96.31% | 2316767 | 3177101 | 91.25% | 5.06% |
| MP1 | 44459358 | 43391063 | 97.6% | 1068295 | 4145172 | 88.28% | 9.32% |
| MP2 | 41627552 | 40819081 | 98.06% | 808471 | 2911060 | 91.07% | 6.99% |
| MP3 | 45769398 | 44854701 | 98% | 914697 | 3258580 | 90.88% | 7.12% |
| MP4 | 44091572 | 43188905 | 97.95% | 902667 | 4265988 | 88.27% | 9.68% |
| MP5 | 73542878 | 71120428 | 96.71% | 2422450 | 5722666 | 88.93% | 7.78% |
| MD1 | 45688724 | 44104325 | 96.53% | 1584399 | 2331199 | 91.43% | 5.1% |
| MD2 | 46990088 | 45357728 | 96.53% | 1632360 | 2409252 | 91.4% | 5.13% |
| MD3 | 59557216 | 56758940 | 95.3% | 2798276 | 5495748 | 86.07% | 9.23% |
| MD4 | 44340734 | 42702890 | 96.31% | 1637844 | 2216928 | 91.31% | 5% |
| MD5 | 41368934 | 39975745 | 96.63% | 1393189 | 2101049 | 91.55% | 5.08% |
| NH1 | 40145966 | 39251110 | 97.77% | 894856 | 3110832 | 90.02% | 7.75% |
| NH2 | 41309306 | 40449569 | 97.92% | 859737 | 3140002 | 90.32% | 7.6% |
| NH3 | 45261606 | 44237647 | 97.74% | 1023959 | 2762257 | 91.64% | 6.1% |
| NH4 | 44053686 | 43133288 | 97.91% | 920398 | 2767656 | 91.63% | 6.28% |
| NH5 | 43276628 | 42308717 | 97.76% | 967911 | 2630896 | 91.68% | 6.08% |
| NP1 | 45430684 | 44489909 | 97.93% | 940775 | 3115254 | 91.07% | 6.86% |
| NP2 | 45901406 | 44267602 | 96.44% | 1633804 | 2308145 | 91.41% | 5.03% |
| NP3 | 44010266 | 42470599 | 96.5% | 1539667 | 2355126 | 91.15% | 5.35% |
| NP4 | 42594310 | 41188421 | 96.7% | 1405889 | 2173906 | 91.6% | 5.1% |
| NP5 | 43477638 | 42031319 | 96.67% | 1446319 | 2191597 | 91.63% | 5.04% |
| ND1 | 41138426 | 39687798 | 96.47% | 1450628 | 2059498 | 91.46% | 5.01% |
| ND2 | 47369546 | 45749971 | 96.58% | 1619575 | 2469086 | 91.37% | 5.21% |
| ND3 | 45436226 | 43823978 | 96.45% | 1612248 | 2351198 | 91.28% | 5.17% |
| ND4 | 43762642 | 42273736 | 96.6% | 1488906 | 2299115 | 91.35% | 5.25% |
| ND5 | 43353484 | 41928317 | 96.71% | 1425167 | 2221639 | 91.59% | 5.12% |

Note: C means Control group, M means Model group, N means taVNS group; H & P & D (HIP & mPFC & ACC).
